# 3-D reconstruction of rice leaf tissue for proper estimation of surface area of mesophyll cells and chloroplasts facing intercellular airspaces from 2-D section images

**DOI:** 10.1093/aob/mcac133

**Published:** 2022-10-25

**Authors:** Rachana Ouk, Takao Oi, Daisuke Sugiura, Mitsutaka Taniguchi

**Affiliations:** Graduate School of Bioagricultural Sciences, Nagoya University, Nagoya 464-8601, Japan; Graduate School of Bioagricultural Sciences, Nagoya University, Nagoya 464-8601, Japan; Graduate School of Bioagricultural Sciences, Nagoya University, Nagoya 464-8601, Japan; Graduate School of Bioagricultural Sciences, Nagoya University, Nagoya 464-8601, Japan

**Keywords:** Chloroplast, curvature correction factor, mesophyll cell, oblate, prolate, *S*_c_, serial section light microscopy (ssLM), *S*_mes_

## Abstract

**Background and Aims:**

The surface area of mesophyll cells (*S*_mes_) and chloroplasts (*S*_c_) facing the intercellular airspace (IAS) are important parameters for estimating photosynthetic activity from leaf anatomy. Although *S*_mes_ and *S*_c_ are estimated based on the shape assumption of mesophyll cells (MCs), it is questionable if the assumption is correct for rice MCs with concave–convex surfaces. Therefore, in this study, we establish a reconstruction method for the 3-D representation of the IAS in rice leaf tissue to calculate the actual *S*_mes_ and *S*_c_ with 3-D images and to determine the correct shape assumption for the estimation of *S*_mes_ and *S*_c_ based on 2-D section images.

**Methods:**

We used serial section light microscopy to reconstruct 3-D representations of the IAS, MCs and chloroplasts in rice leaf tissue. Actual *S*_mes_ and *S*_c_ values obtained from the 3-D representation were compared with those estimated from the 2-D images to find the correct shape-specific assumption (oblate or prolate spheroid) in different orientations (longitudinal and transverse sections) using the same leaf sample.

**Key Results:**

The 3-D representation method revealed that volumes of the IAS and MCs accounted for 30 and 70 % of rice leaf tissue excluding epidermis, respectively, and the volume of chloroplasts accounted for 44 % of MCs. The shape-specific assumption on the sectioning orientation affected the estimation of *S*_mes_ and *S*_c_ using 2-D section images with discrepancies of 10–38 %.

**Conclusions:**

The 3-D representation of rice leaf tissue was successfully reconstructed using serial section light microscopy and suggested that estimation of *S*_mes_ and *S*_c_ of the rice leaf is more accurate using longitudinal sections with MCs assumed as oblate spheroids than using transverse sections with MCs as prolate spheroids.

## INTRODUCTION

Leaf anatomical characteristics are critical factors determining photosynthetic potential. The photosynthetic rate is positively correlated to the total surface area of mesophyll cells (MCs) facing the intercellular airspace (IAS) (Nobel *et al.*, 1975; Psaras *et al.*, 1996; Terashima *et al.*, 2011; Lehmeier *et al.*, 2017). Maximizing the mesophyll surface area facing the IAS per unit leaf area (*S*_mes_) is an effective way to increase the diffusion pathway of CO_2_ (Terashima *et al.*, 2011). Furthermore, since CO_2_ molecules are diffusive to the chloroplasts where they are assimilated, the arrangement of chloroplasts adjacent to the IAS will facilitate the inward diffusion of CO_2_ (Parkhurst, 1994; Psaras *et al.*, 1996). The chloroplast surface area facing the IAS per unit leaf area (*S*_c_) is considered an essential determinant of CO_2_ uptake from the IAS (von Caemmerer and Evans, 1991; Evans *et al.*, 1994; Tholen *et al.*, 2008). Therefore, the *S*_mes_ and *S*_c_ are crucial factors determining the correlation between leaf structure and photosynthetic features (Tholen *et al.*, 2008; Terashima *et al.*, 2011; Adachi *et al.*, 2013; Giuliani *et al.*, 2013).

The *S*_mes_ and *S*_c_ have been estimated by various methods using 2-D section images. The earliest method to measure *S*_mes_ combined paradermal and transverse sections, followed by shape assumptions using a camera lucida (Turrell, 1936). However, this method requires expertise for handling many sections, which is a complicated and laborious approach (Thain, 1983; Théroux-Rancourt *et al.*, 2017). Another method measured the cell surface area using simple shape assumptions as a cylinder or sphere on a section orientation (Morrod, 1974; Bunce *et al.*, 1977), which often unaccounted curvature and fails to represent the cell shapes. Thain (1983) introduced an easy method to correct the unaccounted curvature, which is the degree of inclination of a tangent to the curve on the cell surface, which tends to be less in the centre of the cell than at the cell periphery. Both palisade and spongy cells in typical dicots are assumed to be prolate and oblate, respectively, based on the average ratio of the major and minor axes of cells (Evans *et al.*, 1994) to calculate the curvature correction factor (*F*) (Thain, 1983). Based on its simplicity and accuracy, Thain’s method has become one of the most commonly used parameters for estimating *S*_mes_ and *S*_c_ based on microscopy images of dicot leaves (Evans *et al.*, 1994; Théroux-Rancourt *et al.*, 2017; Sugiura *et al.*, 2020) and monocot leaves (Scafaro *et al.*, 2011; Adachi *et al.*, 2013; Théroux-Rancourt *et al.*, 2017).

**Fig. 1.**
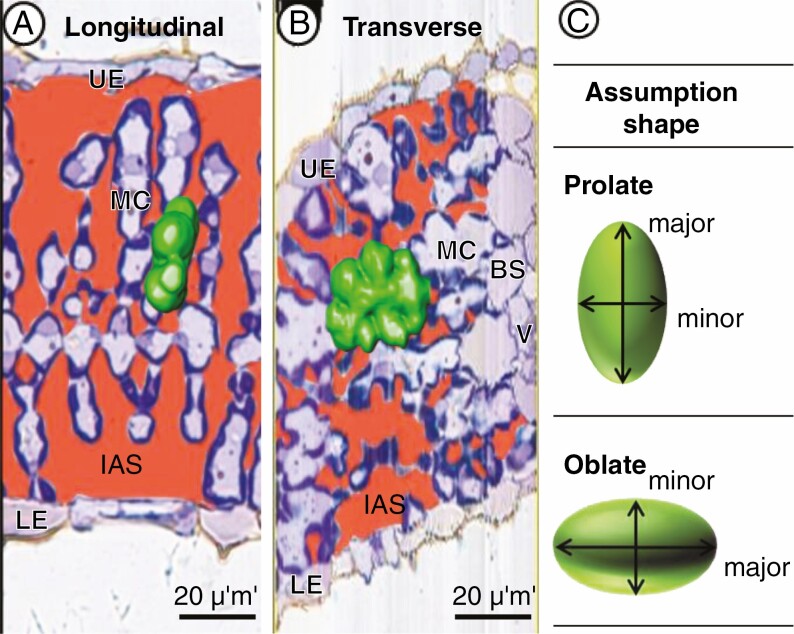
Different structures of rice mesophyll cells according to the sectioning orientation. (A) Longitudinal section. (B) Transverse section. BS, bundle sheath cell; IAS, intercellular airspace; LE, lower epidermis; MC, mesophyll cell; UE, upper epidermis; V, vascular bundle. The green areas in (A) and (B) indicate the same single MC in the 3-D representation. (C) Assumed shape of cells. The major and minor axes are shown in each figure.

The monocot grasses have no distinct differentiation into palisade and spongy cells (Esau, 1977) and have different cell structures depending on whether the sectioning orientation is longitudinal (parallel to the vein) or transverse (perpendicular to the vein), respectively (Chonan, 1970, 1978). Rice is a typical monocot grass, and longitudinal sections of rice leaves show simple MC profiles similar to those of palisade cells in dicot leaves (Fig. 1A) (Esau, 1977), while transverse sections of these show an intricate MC profile similar to that of the spongy cells in the dicot, with more conspicuous lobes on the cell periphery (Fig. 1B) (Chonan, 1967, 1978; Sage and Sage, 2009; Oi *et al.*, 2017; Zhang *et al.*, 2021). For sectioning orientation, the transverse section is used more frequently to show the cell profile as a whole tissue, including epidermal, mesophyll and vascular cells. The *S*_mes_ and *S*_c_ of rice can be calculated from the transverse section, assuming the MCs to be prolate spheroids (Fig. 1C) (Scafaro *et al.*, 2011; Adachi *et al.*, 2013). However, Thain (1983) suggested that the cell shape-specific assumption (for example, as a prolate or oblate spheroid) affected the calculation of the curvature correction factor. Therefore, some estimation errors would occur without carefully assuming the shape-specific features of the cells in the leaf tissue. Thus, it is necessary to apply the correct shape-specific assumptions to estimate the *S*_mes_ and *S*_c_ from the 2-D section accurately.

In order to accurately estimate the *S*_mes_ and *S*_c_ from the 2-D section, it is first necessary to know the actual *S*_mes_ and *S*_c_ values from the 3-D structure. The 3-D reconstruction based on double-stained serial section light microscopy (ssLM) has been established and could successfully detect the deformation of MCs and chloroplasts in rice leaf tissues under salinity stress (Ouk *et al.*, 2020). Therefore, we considered that the ssLM method followed by 3-D reconstruction could be further developed to obtain actual values of *S*_mes_ and *S*_c_ by detecting not only the IAS but also chloroplast regions in serial sections of leaf tissue. Although using micro-focus X-ray computed tomography (micro-CT) is another approach to evaluating *S*_mes_ values from 3-D structures of leaf tissues (Earles *et al.*, 2019), micro-CT cannot detect organelles such as chloroplasts in the cell interior due to the relatively low contrast of X-ray attenuation (Ho *et al.*, 2016; Théroux-Rancourt *et al.*, 2017). On the other hand, ssLM can be used to detect both the IAS and chloroplasts and a light microscope is more accessible than a micro-CT machine. Hence, the ssLM method allows us to compare *S*_mes_ (*S*_c_) values obtained from 3-D representations with those obtained from 2-D section images by applying different shape-specific assumptions (prolate or oblate) and sectioning orientations (transverse or longitudinal) using the same leaf sample.

The aim of this study was (1) to establish a 3-D reconstruction method for the IAS in leaf tissue to obtain the actual *S*_mes*-*3-D_ (*S*_c*-*3D_), and (2) to compare actual *S*_mes*-*3-D_ (*S*_c*-*3D_) from 3-D structure and *S*_mes*-*2D_ (*S*_c*-*2D_) from 2-D section images to find the correct shape-specific assumption that should be used. To establish the 3-D reconstruction of the IAS in addition to MCs and chloroplasts, we further developed our previous ssLM method. To find the correct shape-specific assumption, we used the longitudinal and transverse sections selected from the same 3-D volume rendering data. Since the shape of MCs in rice leaf tissue has not been clearly defined and their concave–convex surface is similar to those of spongy cells in dicots, we hypothesized the shape-specific assumption for rice MCs as oblate spheroids. To test this hypothesis, the curvature correction factor was calculated for prolate or oblate spheroids, and both factors were applied to the conventional estimation of *S*_mes_ and *S*_c_ from 2-D images. Based on these comparisons, we elucidated the proper sectioning orientation and correct shape-specific assumption based on estimation from 2-D images.

## MATERIALS AND METHODS

### Plant materials and growth conditions

Caryopses of rice (*Oryza sativa* L. ‘Nipponbare’) were immersed in distilled water and incubated in a growth chamber at 28/20 °C (day/night). After the white tip of the coleoptile appeared, the seedlings were transplanted on a mesh above a plastic bucket with tap water and grown in a growth chamber under a 14-h photoperiod at 400‒500 µmol m^−2^ s^−1^ and 28/20 °C (day/night). Two days after transplanting, the tap water was changed to nutrient solution (Mae and Ohira, 1981), and all solutions were replaced every 7 d. The seedlings were grown for 25 d.

### Serial sectioning light microscopy

Small segments (1 × 2 mm) from the middle part of fully expanded fifth leaf blades were fixed with Karnovsky’s fixative and osmium tetroxide. Then the segments were dehydrated with acetone, embedded in resin, and the standard methods for transmission electron microscopy were followed (Bozzola and Russell, 1992). The specimen blocks were serially cut, and the sections were observed according to Ouk *et al.* (2020). Longitudinal sections (0.5 µm thickness) placed on a glass slide were double-stained with thionine and acridine orange to stain the chloroplasts and cell walls with deep blue and orange, respectively. The serial sectioning images (image size 1600 × 1200 pixels; pixel size 0.15 µm per pixel; colour depth 24-bit; image file format TIFF) were obtained using a light microscope (BX51, Olympus, Japan) with a CMOS camera (DP74, Olympus).

**Fig. 2.**
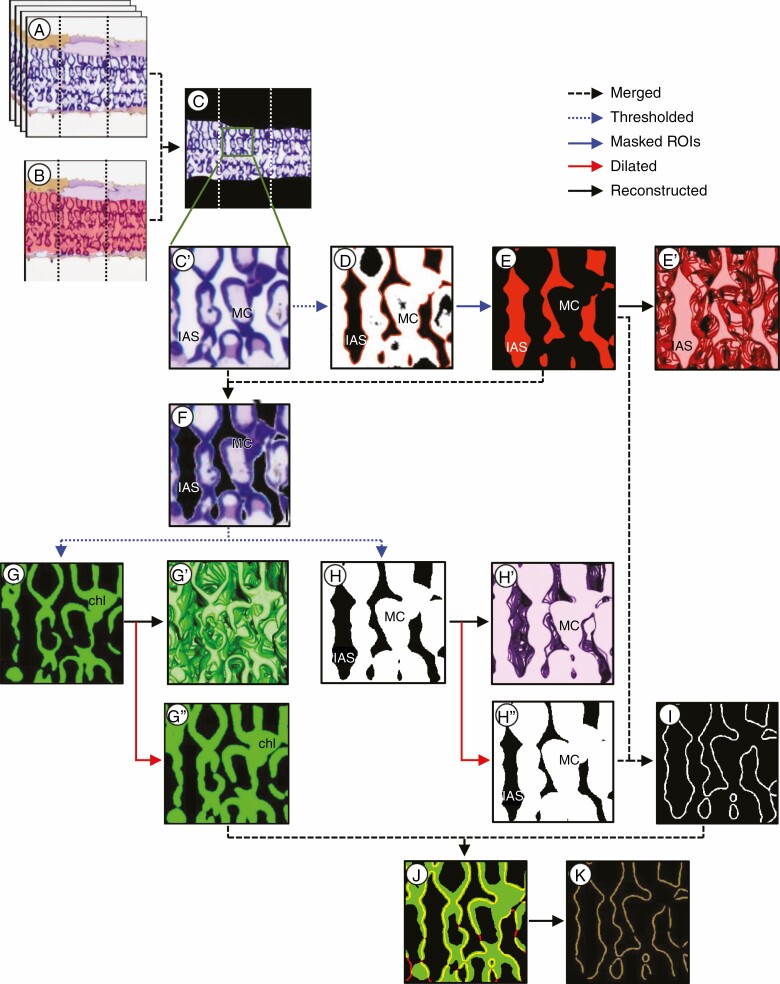
Steps performed to make 3-D reconstructions. (A) Stacked image of longitudinal serial sections. (B) Traced mesophyll tissue. (C) Mesophyll tissue without epidermis. (Cʹ) Magnified image of the box in (C). (D) Thresholded mesophyll tissue and selected IAS as ROIs. (E) Masked ROIs of the IAS. (Eʹ) 3-D reconstruction of the IAS. (F) MC regions without the IAS. (G) Chloroplast regions. (Gʹ) 3-D reconstruction of chloroplast regions. (G″) Dilated chloroplast regions (7 × 7 × 1). (H) Whole MC regions. (Hʹ) 3-D reconstruction of the MC regions. (H″) Dilated MC regions (7 × 7 × 1). (I) Cell wall line by merging the masked ROIs of IAS (E) and dilated MC regions (H″). (J) Overlapped dilated chloroplast regions (green) and cell wall lines (red) to detect chloroplast regions in contact with the cell wall (yellow). (K) Extracted chloroplast regions in contact with cell wall lines facing IAS. The straight dashed lines in (A–C) indicate the areas used for reconstruction and measurement (see Fig. 3). (A–C) are full-size images (1000 × 1000 pixels). (Cʹ–K) are magnified images.

The brightness and contrast of serial section images were adjusted, and the processed images were aligned using the plugin tool ‘Register Virtual Stack Slices’ (Translate, Rigid—translate + rotate) and then cropped (1000 × 1000 pixels) using the plugin tool ‘Crop (3D)’ of Fiji software (http://fiji.sc/Fiji, National Institutes of Health, USA) (Fig. 2A).

### Image processing and 3-D reconstruction

The image stacks were 3-D-reconstructed and image-processed. The IAS and chloroplast regions in contact with the cell wall were extracted and reconstructed into 3-D representations using Image-Pro 3D (ver. 10, Media Cybernetics, USA).

The mesophyll tissue was manually traced as the region of interest (ROI) to exclude the upper and lower epidermis using the 2D segmentation (Active ROI polygon) tool in the software (Fig. 2B) and merged to the aligned images (Fig. 2A). To extract the IAS, the mesophyll tissue (Fig. 2C, Cʹ) was binarized using the thresholding method, and the IAS were binarized using the Magic wand tool by selecting as ROIs (Fig. 2D ), then the selected ROIs were masked (Fig. 2E) and reconstructed in a 3-D representation (Fig. 2Eʹ). The masked ROIs (Fig. 2E) were merged to the mesophyll tissue without epidermis (Fig. 2C, Cʹ) to remove the IAS (Fig. 2F). Then, the mesophyll tissues without the IAS were binarized using the thresholding method to separate chloroplast regions (Fig. 2G) and whole MC regions (Fig. 2H). Then, the chloroplast regions and whole MC regions were reconstructed into separate 3-D representations (Fig. 2Gʹ, Hʹ).

To extract the chloroplast regions in contact with the cell wall, the whole MC regions (Fig. 2H) were dilated using the 3D filters (Dilation, width × height × depth = 7 × 7 × 1) tool (Fig. 2Hʹ) and merged with the masked IAS (Fig. 2E) to detect the overlapped area as cell wall lines (Fig. 2I). Then, the cell wall lines (Fig. 2I) were merged with the dilated chloroplast regions (7 × 7 × 1) (Fig. 2Gʹ) to detect overlapped regions (yellow, Fig. 2J), and then chloroplast regions in contact with the cell wall facing the IAS (Fig. 2K) were extracted.

All of the 3-D surface rendering model were reconstructed with subsampling (512M voxel) and smoothing (low-pass filter [3 × 3 × 3]), and the volume and surface area were calculated using the ‘3D Measure’ tool in the software.

### Calculation of S_*mes*_*and* S_*c*_ from the 3-D reconstruction

The *S*_mes*-*3D_ and *S*_c*-*3D_ were calculated from the modified equations of Evans *et al.* (1994). The surface area of MC facing the IAS per unit leaf area (*S*_mes_) in volume rendering sequence was calculated as follows:


$$\begin{array}{l} S_{mes\text{\textasciitilde{}}\text{-}3D}=\frac{S_{IAS}}{Leaf\;area}\;(\mu m^{2}\mu m^{-2}) \end{array}$$ (1)

where *S*_IAS_ is the surface area of the IAS (Fig. 2Eʹ) (µm^2^). Leaf area (µm^2^) is the product of the width of sections (µm) and the depth of the stacked images (µm).

The surface area of chloroplast regions facing the IAS per unit leaf area (*S*_c_) in volume rendering sequence was calculated as:


$$\begin{array}{l} S_{c\text{\textasciitilde{}}\text{-}3D}=\frac{S_{chl}}{Leaf\;area}\;(\mu m^{2}\mu m^{-2}) \end{array}$$ (2)

where *S*_chl_ is the surface area of chloroplast regions in contact with cell wall lines facing the IAS (Fig. 2K) (µm^2^).

### 2-D method to estimate S_*mes*_ and S_*c*_

**Fig. 3.**
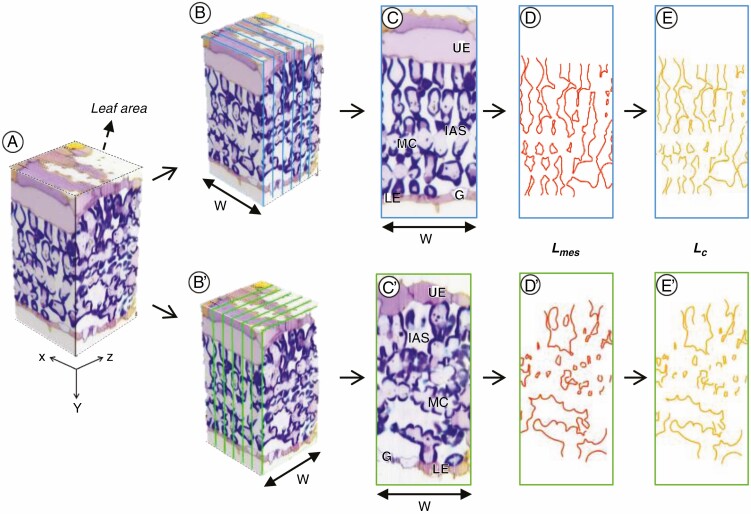
Diagram demonstrating anatomical measurements on the different leaf sectioning orientations. (A) 3-D volume of stacked serial images. (B‒E) Longitudinal sections. (Bʹ‒Eʹ) Transverse sections. (B, Bʹ) Selected images from serial sequences. (C, Cʹ) Selected image. (D, Dʹ) Total peripheral length of MCs facing the IAS (*L*_mes_). (E, Eʹ) Total peripheral length of chloroplasts facing the IAS (*L*_c_). The blue and green lines in B and Bʹ indicate image numbers. LE, lower epidermis; G, guard cell; UE, upper epidermis; W, width of section used for the measurement (55 µm). Cutting interval (*z*-steps) = 0.5 µm. Total number of cuttings = 110.

The serial images were stacked and cropped to adjust the same width (*xy* and *zy* directions) using the Volume of interest tool in Image-Pro 3D (Fig. 3A). Six sections were selected from both the longitudinal (Fig. 3B) and transverse (Fig. 3Bʹ) orientations. The MCs (Fig. 3D, Dʹ) and the chloroplasts (Fig. 3E, Eʹ) facing the IAS per unit leaf area were segmented using the Magic wand tool and were traced on a new layer using Photoshop software (ver. CS5, Adobe, USA). The total peripheral lengths of the MCs and chloroplasts facing the IAS (*L*_mes_ and *L*_c_, respectively; µm) were computed by Fiji software.

We calculated *S*_mes*-*2D_ as proposed by Evans *et al.* (1994):


$$\begin{array}{l} S_{mes\text{\textasciitilde{}}\text{-}2D}=\frac{L_{mes}}{W}\;\times \;F\;\;\;\;\;\;\;\;\;\;\;\;(\mu m^{2}\mu m^{-2}) \end{array}$$ (3)

where *W* is the width of a section (µm). *F* is the curvature correction factor calculated from the shape-specific assumption aspect ratios of the prolate (*F*_p_) as the minor to major axis and the oblate (*F*_o_) as the major to minor axis on each sectioning orientation (Fig. 3) as described previously (Thain, 1983; Evans *et al.*, 1994) (Supplementary Data Table S1). The major and minor axes of cells from longitudinal (10–17 cells) and transverse (3–8 cells) sections were measured using the Fiji software.

We calculated *S*_c*-*2D_ using the following equation:


$$\begin{array}{l} S_{c\text{\textasciitilde{}}\text{-}2D}=\frac{L_{c}}{W}\;\times \;S_{mes\text{-}2D}\;\;\;\;\;\;\;\;\;\;\;\;(\mu m^{2}\mu m^{-2}) \end{array}$$ (4)

where *L*_c_ is the total peripheral length of chloroplasts facing the IAS. Hereafter, the numerical calculation from 2-D section images is referred to as ‘2-D estimation’.

### Statistical analysis

Three plants were the replication, and one leaf segment was taken per plant. The data were statistically analysed using Microsoft Excel for Windows with the add-in software Statcel 3 (OMS Publishing, Japan).

## RESULTS AND DISCUSSION

### 3-D approach to obtaining leaf anatomy properties, S_*mes*_ and S_*c*_

**Fig. 4.**
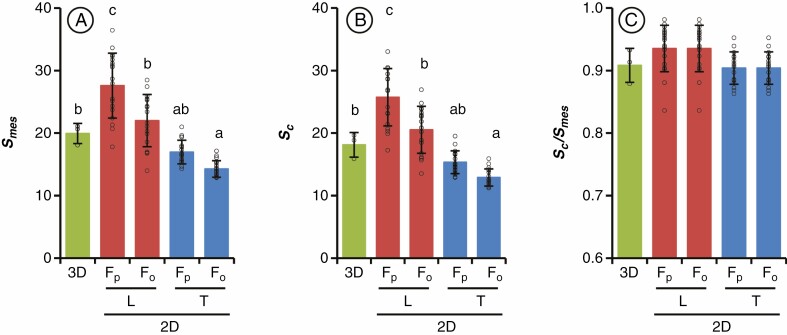
Comparison of the surface area of mesophyll cells and chloroplast regions facing the IAS obtained from 3-D reconstruction-based calculation and 2-D estimation with different leaf sectioning orientations. (A) Mesophyll surface area facing the IAS (*S*_mes_). (B) Surface area of chloroplasts facing the IAS (*S*_c_). (C) Ratio of *S*_c_ to *S*_mes_ in the whole leaf area. *S*_mes_ and *S*_c_ in transverse (T) and longitudinal (L) sections were estimated with the correction curvature factors assumed for a prolate (*F*_p_) or oblate (*F*_o_) spheroid. Mean ± standard deviation (*n* = 3). Circle points represent individual data of 3-D (*n* = 3) and 2-D (*n* = 6 sections × 3 plants). Different letters indicate significant differences among the measurements compared by Tukey–Kramer multiple comparison tests (*P* < 0.05, *n* = 3).

The ssLM method produced image sequences with sufficient contrast to distinguish MCs, chloroplasts, and the IAS (Figs 2 and 3, Supplementary Data Videos S1 and S2). The stacking of the serial section images and the subsequent 3-D reconstruction visualized the connection of IAS and detected the mesophyll chloroplasts in the whole leaf tissues (Supplementary Data Videos S1 and S2). Based on the 3-D reconstruction, the volumes of leaf tissue (excluding epidermis), IAS, MCs, and chloroplasts were calculated (Table 1). The results showed that IAS accounted for 29.8 % of the total volume of leaf tissue (Table 1). The calculated *S*_mes-3D_ was ~20 µm^2^ µm^‒2^ (Fig. 4A), higher than the values obtained from 2-D transverse sections using the same rice genotype grown under low and high light intensity, which was reported to affect leaf thickness and influence *S*_mes_ (Ellsworth *et al.*, 2018).

**Table 1.** Anatomical parameters of leaf tissue based on 3-D reconstructions.

Parameter	IAS	MCs	Chloroplasts
Volume per leaf area (µm^3^ m^‒2^)	24.27 ± 2.67	49.60 ± 8.05	22.65 ± 2.67
Volume ratio in tissue (%)	29.77 ± 5.08	70.55 ± 5.39	32.20 ± 1.29

Values are mean ± s.d. (*n* = 3).

Although light microscopy cannot determine the morphology of individual chloroplasts in MCs due to lower resolution compared with electron microscopy, the present result indicated that the total volume of chloroplasts was 32.2 % in tissue (Table 1), equivalent to 44.0 % of MCs, which was comparable to the previous study in rice (47 %; Oi *et al.*, 2020) and higher than that in wheat (32 %; Harwood *et al.*, 2020). The greater chloroplast volume in MCs allows higher contents of photosynthetic proteins per volume in rice leaves (Sage and Sage, 2009; Adachi *et al.*, 2013). Our 3-D reconstruction of chloroplast regions further extends the capability to calculate the *S*_c_, which is more directly related to the photosynthetic rate and mesophyll conductance (Evans *et al.*, 1994; Tholen *et al.*, 2008). The concept for 3-D detection of the surface area of chloroplast regions in contact with cell wall lines facing the IAS (Fig. 2J, K) was derived from the length of the chloroplast periphery facing the IAS in 2-D sections (Evans *et al.*, 1994). It should be highlighted that the sections in this study (500 nm) were thicker than those obtained by focused ion beam (FIB)-SEM and serial block-face (SBF)-SEM (50 nm, Oi *et al.*, 2017; Harwood *et al.*, 2020). Therefore, the *S*_c_ calculated using ssLM would be overestimated compared with using high-resolution electron microscopy. However, the improved method of obtaining the overlapped area in 3-D representations can detect the chloroplast surface area in contact with the cell wall (Fig. 2G–K) and shed light on understanding of the connection between 3-D leaf anatomy and photosynthetic traits. To the best of our knowledge, this is the first report proposing a method to compute the *S*_c_ value from a 3-D reconstruction. Methods for analysing leaves in 3-D representation are critical for developing the ‘ideal’ leaf, which is defined as balancing surface area for gas exchange to maximize photosynthesis (McCormick, 2017).

### 2-D sectioning orientation and shape-specific assumption to estimate S_*mes*_ and S_*c*_

Due to the high resolution of 3-D volume rendering stacked from longitudinal sections, the reconstructed transverse sections also showed leaf structure in detail (Fig. 3A, Bʹ, Cʹ). The rice MCs showed different shapes depending on the sectioning orientations; the uniformed oblong shape of MCs in the longitudinal sections (Fig. 3B) formed a vertically narrow IAS (Figs 1A and 3C), whereas the intricate shape of MCs with a number of lobes in transverse sections (Fig. 3Bʹ) formed a horizontally concave IAS (Figs 1B and 3Cʹ). The anatomical measurement in the 2-D sections showed that *L*_mes_ and *S*_mes*-*2D_ were significantly higher in the longitudinal section than in the transverse section (Table 2, Fig. 4). The shape of MCs also affected the curvature correction factors, with significant differences depending on the sectioning orientation (Table 2). The oblong shape of the rice MCs in the longitudinal and paradermal sections (Ouk *et al.*, 2020) appears similar to that of palisade cells of dicot leaves (Esau, 1977); however, the concave–convex contours of rice MCs sometimes make them look like spongy cells (Esau, 1977; Zhang *et al.*, 2021). Therefore, the curvature correction factors with the assumption of a prolate (*F*_p_) or oblate (*F*_o_) spheroid were calculated in both the longitudinal and transverse sections (Table 2) and were applied to calculate *S*_mes_ in the 2-D images (Fig. 4A). The highest *S*_mes*-*2D_ value was obtained when *F*_p_ was applied to the longitudinal sections, and the lowest *S*_mes*-*2D_ value was obtained when *F*_o_ was applied to the transverse sections.

**Table 2.** Anatomical parameters of leaf tissue estimated from 2-D section images.

Parameter	Longitudinal	Transverse
*L*_mes_ (µm)	1068 ±** **115.52**	673 ±** **27.13
*L*_c_(µm)	996 ±** **110.08**	609 ±** **28.30
*F*_p_	1.42 ±** **0.02*	1.38 ±** **0.02
*F*_o_	1.13 ±** **0.01*	1.17 ±** **0.02

*F*_p_ and *F*_o_ are the curvature correction factors calculated from the cells (*n* = 10–17, 3–8) in longitudinal and transverse sections assumed for prolate and oblate spheroids, respectively. The width of the sections was adjusted to the same length (55 µm).

Values are mean ± s.d. (*n* = 3 for biological replication averaged from six sections).

Asterisks indicate a significant difference between longitudinal and transverse sections compared by Student’s *t*-test (**P < 0.01, *P < 0.05). Values are mean ± s.d. (*n* = 3 for biological replication averaged from six sections).

Compared with the value obtained from the 3-D representation, the 2-D estimation from the longitudinal sections tended to overestimate while the transverse sections tended to underestimate (Fig. 4), even though both sectioning orientations were selected from the same 3-D volume rendering. Moreover, the curvature correction factor of *F*_o_ seemed to fit better for the rice MCs than *F*_p_ in the longitudinal sections, possibly because the actual shape of MCs was closer to an oblate spheroid (Fig. 1C). The *S*_mes*-*2-D_ values estimated using the longitudinal sections with *F*_o_ were close to the *S*_mes*-*3D_ without significant difference (Fig. 4A), with 10 % discrepancy between *S*_mes*-*3D_ and *S*_mes*-*_2-D, similar to the previous study using X-ray micro-CT (Théroux-Rancourt *et al.*, 2017). Our comparative results suggest that the discrepancy degree between *S*_mes_ values obtained from the 3-D reconstruction-based calculation and the 2-D estimation depends on the shape-specific assumption of the curvature correction factors on the sectioning orientations (Fig. 4), besides the section thickness and numbers (Théroux-Rancourt *et al.*, 2017). Rice, a monocotyledonous grass with thin leaves and parallel veins, also showed a high deviation between 2-D estimation and 3-D reconstruction-based calculation (Mathers *et al.*, 2018). This could be due to the sectioning orientation of the rice leaves, which differed from that of dicot leaves showing similar MC contours in both longitudinal and transverse sections. In our study, the estimation based on the sectioning orientations and cell shape assumption caused discrepancies of 10‒38 %; in particular, using transverse sections of rice leaves tended to underestimate *S*_mes_ (Fig. 4A). These results suggest that using *F*_o_ for the longitudinal section in 2-D images can be considered optimal for an accurate estimation of *S*_mes_ and *S*_c_ in rice leaves.

The chloroplasts appeared to be narrow and adjacent to the cell wall of the MCs in both longitudinal and transverse sections (Fig. 3). The *L*_*c*_ was measured from the distribution of the chloroplast surface adjacent to the MC wall. Although the *L*_*c*_ was significantly lower than the *L*_mes_ in both longitudinal and transverse sections (Table 1), trends in estimated *S*_c_ values among the 3-D and 2-D image analyses (Fig. 4B) were similar to *S*_mes_ trends (Fig. 4A). The *S*_c_/*S*_mes_ ratio did not differ significantly between the 2-D and 3-D image analyses (Fig. 4C), suggesting that the *S*_c_/*S*_mes_ value is less affected by sectioning orientation as the chloroplasts of rice MCs cover almost all of the cell periphery and fit the shape of MCs (Sage and Sage, 2009; Oi *et al.*, 2017; Ouk *et al.*, 2020).

The *S*_c_ value has been known to be closely related to photosynthetic parameters (Evans *et al.*, 1994; Tholen *et al.*, 2008). The *S*_c *-*3D_ obtained by the ssLM method strengthens the detection of chloroplast deformation under stress conditions. For example, in rice under salinity stress, the chloroplasts altered their shape from flat to round (Oi *et al.*, 2020), resulting in a decreased coverage area of chloroplasts on the cell periphery (Ouk *et al.*, 2020) and possible change in *S*_c_ values. Therefore, the relationship between changes in leaf anatomy (chloroplast structure) and photosynthetic capacity under stress conditions will be investigated by the ssLM method in future work.

## CONCLUSIONS

Serial section light microscopy followed by 3-D reconstruction can visualize the IAS and detect chloroplast regions in the whole leaf tissue. The obtained results will link the morphological characteristics of the IAS, MCs and chloroplasts with the photosynthetic properties of leaves. Furthermore, this method revealed differences in the 2-D estimation of the *S*_mes_ and *S*_c_ caused by the orientation of leaf sectioning and the application of curvature correction factors. We conclude that using the longitudinal section with shape-specific assumption of oblate spheroids is the best method to determine the *S*_mes_ and *S*_c_ more easily with only 2-D images. The 2-D method is as accurate as 3-D representations regardless of sectioning orientation in the calculation of *S*_c_ /*S*_mes_. The resolution of light microscopy is sufficient for organelle detection, which is not possible with micro-CT, and can help to elucidate fundamental questions about the structural connections from intercellular to intracellular in leaf tissues.

## SUPPLEMENTARY DATA

Supplementary data are available online at https://academic.oup.com/aob and consist of the following:

Video S1: 3-D reconstruction of IAS in rice leaf tissue. Cutting interval (*z*-steps) = 0.5 µm. Total number of cuttings = 110.

Video S2: 3-D reconstruction of chloroplasts and chloroplast regions in contact with cell wall lines facing the IAS in rice leaf tissue. Green and yellow colours correspond to chloroplasts and chloroplast regions in contact with cell wall lines facing the IAS, respectively. Cutting interval (*z*-steps) = 0.5 µm. Total number of cuttings = 110.

Table S1: shape assumption to estimate the curvature correction factors of rice mesophyll cells.

mcac133_suppl_Supplementary_Table_S1

mcac133_suppl_Supplementary_Video_S1

mcac133_suppl_Supplementary_Video_S2
