# Supplementary material for: 3-D reconstruction of rice leaf tissue for proper estimation of surface area of mesophyll cells and chloroplasts facing intercellular airspaces from 2-D section images
Source: Ann Bot. 2022 Oct 25;130(7):991–8. doi: 10.1093/aob/mcac133 (PMC9851327; doi:10.1093/aob/mcac133)
Supplement: mcac133_suppl_Supplementary_Table_S1 [file mcac133_suppl_supplementary_table_s1.docx]

**Table S1. The shape assumption to estimate the curvature correction factors of rice mesophyll cells.**

Oblate

Prolate

Sphere

| Assumption shape | Aspect ratio | Curvature correction factors | |
| --- | --- | --- | --- |
|  |  | Prolate spheroid | Oblate  spheroid |
| 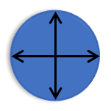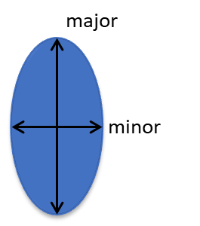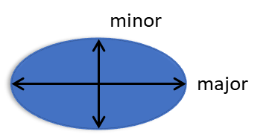 | 0.0 | 1.57 |  |
|  | 0.1 | 1.55 |  |
|  | 0.2 | 1.52 |  |
|  | 0.4 | 1.45 |  |
|  | 0.6 | 1.38 |  |
|  | 0.8 | 1.32 |  |
|  | 1.0 | 1.27 | 1.27 |
|  | 1.5 |  | 1.19 |
|  | 2.0 |  | 1.14 |
|  | 3.0 |  | 1.08 |
|  | 4.0 |  | 1.05 |
|  | 6.0 |  | 1.03 |
|  | 10.0 |  | 1.01 |

Note: The assumption is derived from Thain (1983). The aspect ratios for prolate and oblate are ratios of minor-to-major axis and of major-to-minor axis, respectively.
